# Synergy conformal prediction applied to large-scale bioactivity datasets and in federated learning

**DOI:** 10.1186/s13321-021-00555-7

**Published:** 2021-10-02

**Authors:** Ulf Norinder, Ola Spjuth, Fredrik Svensson

**Affiliations:** 1 Department of Pharmaceutical Biosciences and Science for Life Laboratory, Uppsala University, Box 591, SE-75124 Uppsala, Sweden; 2 Department of Computer and Systems Sciences, Stockholm University, Box 7003, 164 07 Kista, Sweden; 3 MTM Research Centre, School of Science and Technology, Örebro University, 70182 Örebro, Sweden; 4 Alzheimer’s Research UK UCL Drug Discovery Institute, University College London, The Cruciform Building, Gower Street, London, WC1E 6BT UK

**Keywords:** Conformal prediction, Federated learning, Confidence, Machine learning

## Abstract

Confidence predictors can deliver predictions with the associated confidence required for decision making and can play an important role in drug discovery and toxicity predictions. In this work we investigate a recently introduced version of conformal prediction, synergy conformal prediction, focusing on the predictive performance when applied to bioactivity data. We compare the performance to other variants of conformal predictors for multiple partitioned datasets and demonstrate the utility of synergy conformal predictors for federated learning where data cannot be pooled in one location. Our results show that synergy conformal predictors based on training data randomly sampled with replacement can compete with other conformal setups, while using completely separate training sets often results in worse performance. However, in a federated setup where no method has access to all the data, synergy conformal prediction is shown to give promising results. Based on our study, we conclude that synergy conformal predictors are a valuable addition to the conformal prediction toolbox.

**Supplementary Information:**

The online version contains supplementary material available at 10.1186/s13321-021-00555-7.

## Introduction

Confidence predictors [1], such as conformal predictors, have been demonstrated to have several properties that make them useful for predictive tasks in drug discovery and other biomedical research [2]. Well calibrated models with defined uncertainties can facilitate decision making and has been identified as an important area of development [3, 4].

Conformal predictors allow predictions to be made at a pre-set confidence level, with errors guaranteed to not exceed that level. This is achieved under only mild conditions. Both transductive [5] and inductive conformal predictors [6] (ICP) have been described but we will focus on ICP in this study. The basis of an ICP is that a calibration set is used to relate new predictions to calibration instances with known labels. The conformal predictor then outputs a prediction region based on the calibration results and the selected confidence level. For example, a prediction set for a binary classification has four possible outcomes, no prediction, either of the two labels, or both labels. For details on how this is achieved we direct the reader to Norinder et al*.* [7] and Alvarsson et al. [8]. Reviews on the application of conformal prediction in the field of cheminformatics are also available [2, 3]. Conformal predictors can be calibrated for each class separately, called Mondrian conformal predictors. Mondrian conformal predictors have been shown not only to give the expected error rate for each class independently, but also to give excellent performance for imbalanced data [9, 10].

When evaluating conformal predictors two key metrics are validity and efficiency. Validity measures the fraction of predictions containing the correct label while efficiency measures the fraction of predictions containing only one label (or in the case of regressions, the width of the prediction region). The properties of conformal prediction guarantees that validity is always achieved as long as the conditions are met. It is generally desired to have as high efficiency as possible to maximise the utility of the predictions.

Several different approaches have been described for conformal prediction. The baseline ICP method uses fixed predefined training and calibration sets. Commonly, this process is repeated multiple times with different splits between training and calibration, and the p-values averaged, in what is called an aggregated conformal predictor (ACP) [11, 12]. This has the advantage that the prediction becomes less sensitive to the split between training and calibration data. However, while ACPs empirically have been shown in many applications to generate valid conformal predictors (an error rate not exceeding the set confidence-level) [13, 14], they have not been theoretically proven to be valid.

Recently, a new type of conformal predictor, called a synergy conformal predictor (SCP), has been introduced for classification [15] and regression problems [16]. In this application, the nonconformity scores from several different predictors are aggregated to construct a conformal predictor using a shared calibration set. This approach has been shown to satisfy the requirements for theoretical validity. SCP has previously been applied to toxicity predictions [17], but applications to other cheminformatics problems have to our knowledge not been reported and a systematic evaluation of SCP in cheminformatics is not available.

**Fig. 1:**
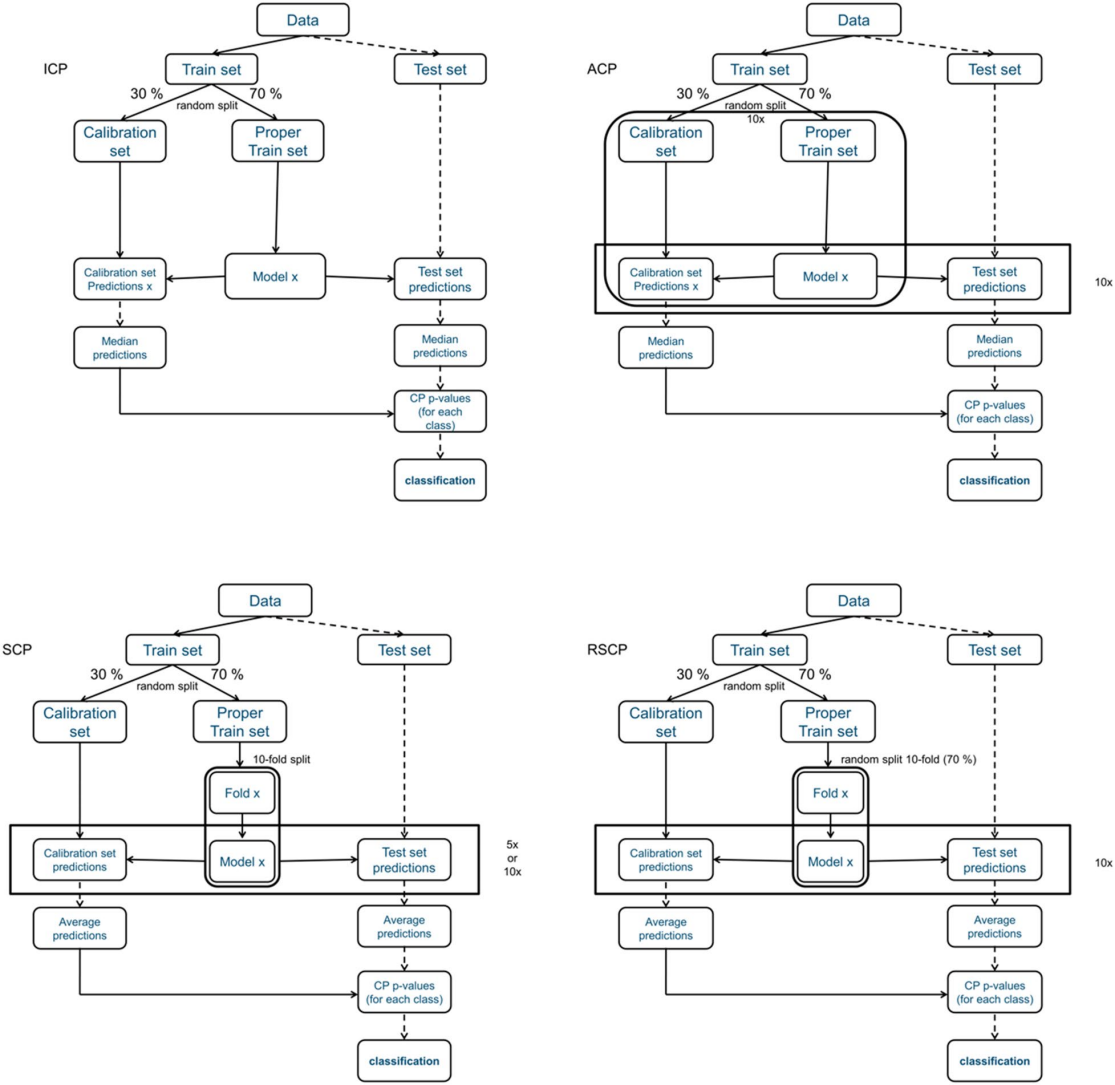
Outline of the different conformal prediction algorithms used in this study. Split percentages and number of repeats reflect the methods used in this study. The difference between the algorithms lies in the way the data is split. Note that an ICP is equivalent to an ACP with just one split

Key aspects of the different conformal predictors are shown schematically in Fig. 1. While the basic principle remains the same, the key difference between the different conformal predictors is the strategy used to split the data. Splitting the training data into smaller individual sets for SCP risk decreasing the predictive performance of the model compared to approaches trained on the full training set. However, the disjoint training sets allow for applications in for example federated learning [18] or distributed training that is not possible to achieve with other conformal methods that require access to all the available training data.

Federated learning is the process where several parties jointly train a machine learning model but keep their respective data local and private [19]. Federated learning can therefor help overcome issues related to confidentiality or privacy of data while still generated models based on a large amount of data.

Previous work has shown that prediction intervals from multiple non-disclosed datasets can be integrated by aggregating conformal p-values, but without producing valid results [20]. Applying SCP for federated learning is also convenient as it is a rigorously defined framework for aggregating the results from multiple sources. However, the aggregation still requires access to a shared calibration set.

SCP can also be used to construct predictor ensembles with overlapping training data as long as the calibration set remains the same. This allows for each split to contain sufficient training data to generate well-performing models regardless of the number of splits used and might allow for more efficient models compared to a single ICP predictor while still maintaining the guaranteed error rate as SCP methods have been shown to be theoretically valid.

In this study, we compare the performance of SCP with that of ICP and ACP on large-scale bioactivity datasets. We also explore potential applications of SCP in federated learning.

## Results and discussion

**Fig. 2:**
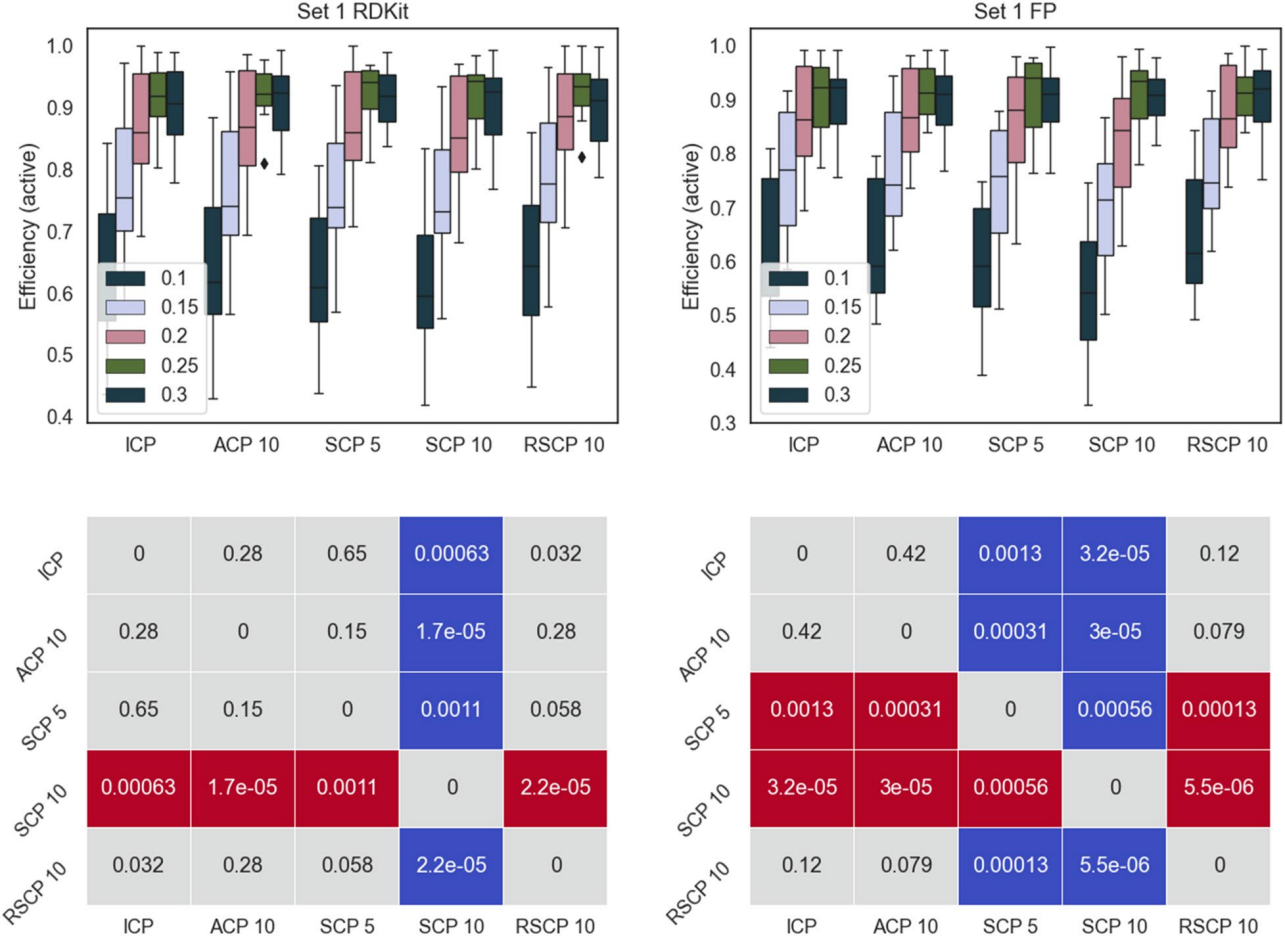
Top panels: efficiency for the active class for Set 1 using the different conformal predictors at a range of significance levels (0.1–0.3). Results for RDKit descriptors left and fingerprints right. Bottom panels: pairwise comparison (Wilcoxon signed-rank test with Bonferroni correction for multiple testing, 0.05 significance level, across all significance levels and datasets) of methods on rows with methods on columns, significantly better result is indicated in blue, significantly worse result in red. p-values are indicated in the figure. For example, in the bottom left panel we can see that SCP 10 is significantly worse than all other methods it is compared with

**Fig. 3:**
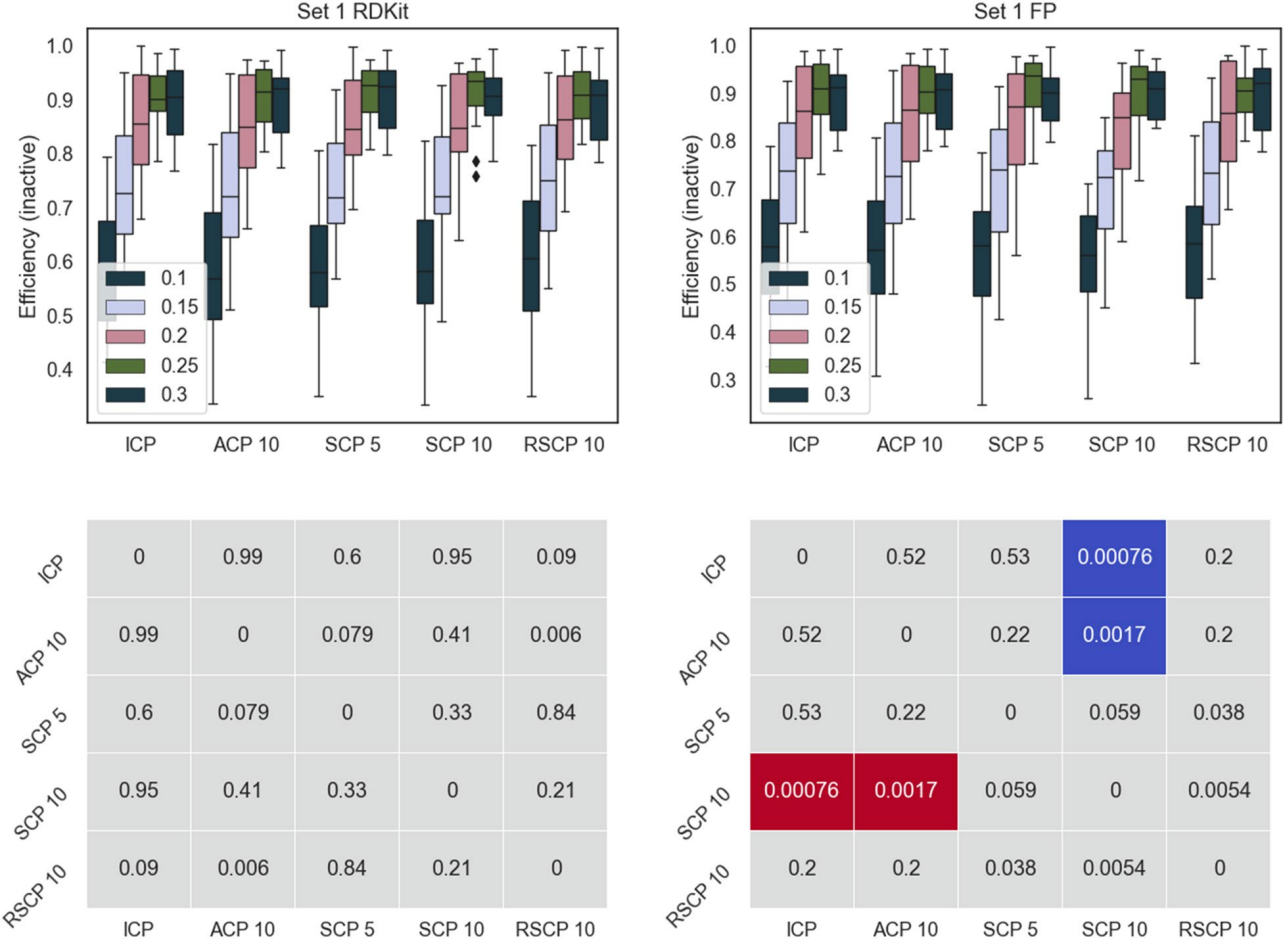
Top panels: efficiency for the inactive class for Set 1 using the different conformal predictors at a range of significance levels (0.1–0.3). Results for RDKit descriptors left and fingerprints right. Bottom panels: pairwise comparison (Wilcoxon signed-rank test with Bonferroni correction for multiple testing, 0.05 significance level, across all significance levels and datasets) of methods on rows with methods on columns, significantly better result is indicated in blue, significantly worse result in red. p-values are indicated in the figure

**Fig. 4:**
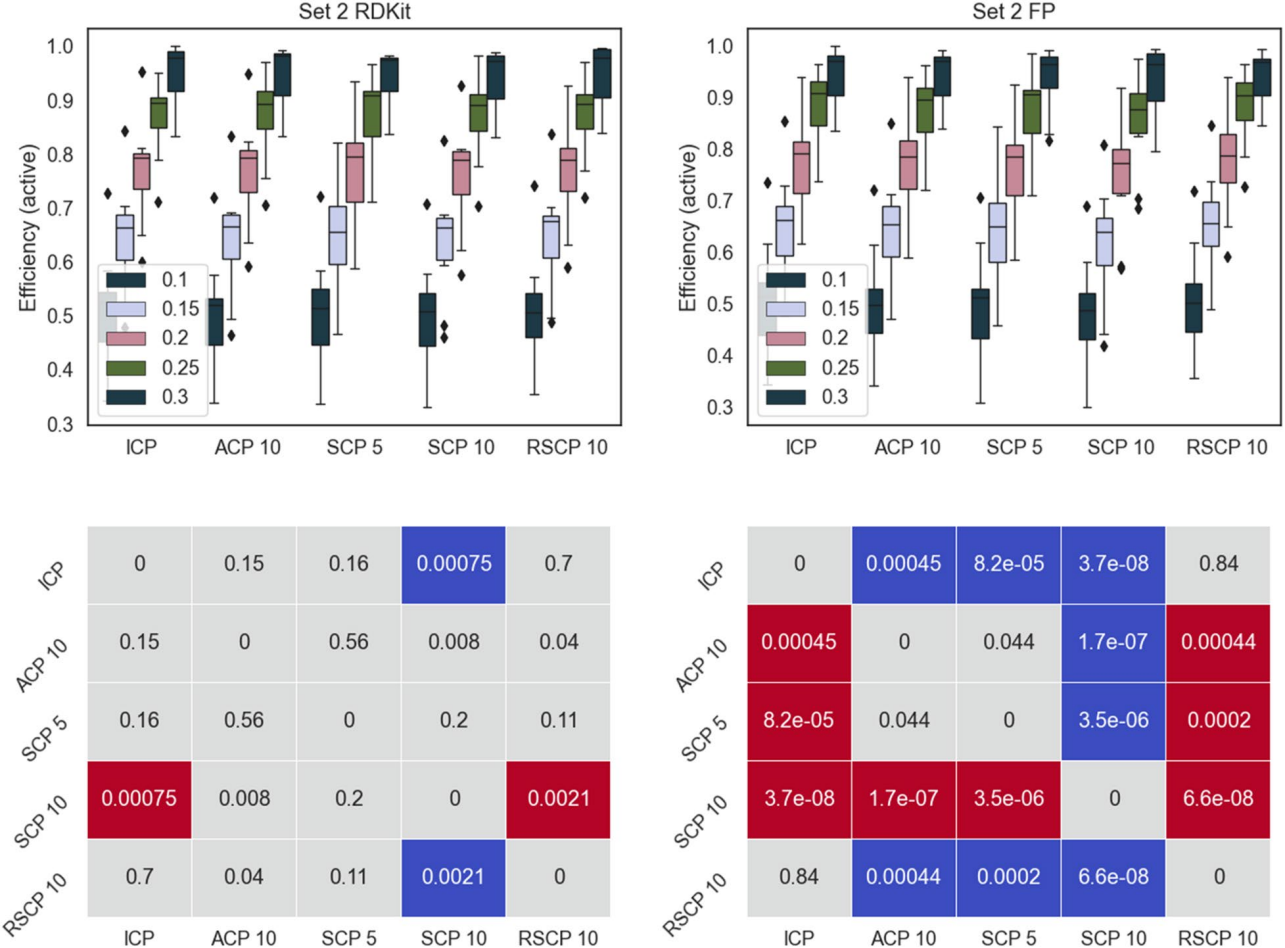
Top panels: efficiency for the active class for Set 2 using the different conformal predictors at a range of significance levels (0.1–0.3). Results for RDKit descriptors left and fingerprints right. Bottom panels: pairwise comparison (Wilcoxon signed-rank test with Bonferroni correction for multiple testing, 0.05 significance level, across all significance levels and datasets) of methods on rows with methods on columns, significantly better result is indicated in blue, significantly worse result in red. p-values are indicated in the figure

**Fig. 5:**
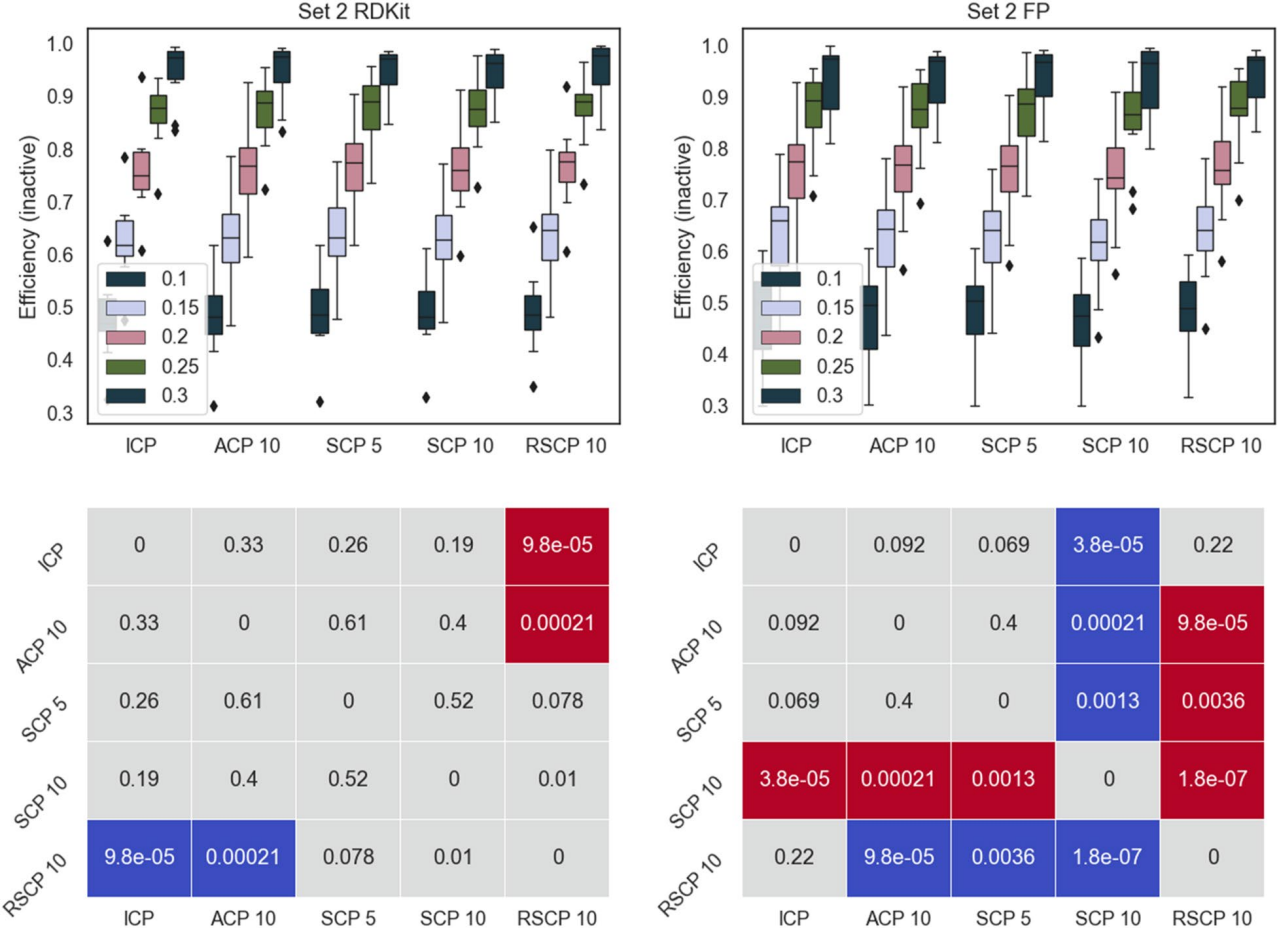
Top panels: efficiency for the inactive class for Set 2 using the different conformal predictors at a range of significance levels (0.1–0.3). Results for RDKit descriptors left and fingerprints right. Bottom panels: pairwise comparison (Wilcoxon signed-rank test with Bonferroni correction for multiple testing, 0.05 significance level, across all significance levels and datasets) of methods on rows with methods on columns, significantly better result is indicated in blue, significantly worse result in red. p-values are indicated in the figure

To evaluate SCP for bioactivity data, two sets of PubChem data described by two sets of descriptors were used. These datasets have previously been used for machine learning evaluations [21, 22]. We compared the performance of SCP with five or ten splits (SCP 5 and SCP 10), SCP with ten random overlapping splits (RSCP 10), ACP with ten aggregations (ACP 10), and ICP. The results were evaluated using mainly the model efficiency, defined as the fraction of single label predictions. This is due to the fact that we expect all conformal predictors to give valid models, that is models with an error rate corresponding to the set significance level. See the methods section for more detail on these metrics. Efficiency for all methods is shown Figs. 2, 3, 4, 5 along with pairwise comparison for statistically significant differences (Wilcoxon signed-rank test). All methods produced valid models (see Additional files 1 and 2).

Overall, all the methods follow a similar pattern for the efficiencies and there are no dramatic differences, this is also evident from the fact that most of the comparisons did not produce a statistically significant difference in performance. However, ICP and RSCP tend to deliver slightly more efficient models at the higher confidence levels. This can be rationalized by ACPs tendency to produce slightly over valid models (overconservative) with a resulting loss in efficiency. For SCP 5 and SCP 10, the division of the training data is likely the cause of the lower efficiency, this is also supported by the overall lower efficiency for SCP 10.

Despite the somewhat lower efficacy of the SCP models, our results indicate that they can still generate well-performing models. Especially when not dividing the training data in too many partitions, as seen from the generally better performance of SCP 5 compared to SCP 10. In situations where a single joint training set is not available, either for technical reasons (aggregating a large amount of for example image data might be challenging), or where data cannot be shared between collaborators for reasons of confidentiality, SCP can be an option where models can be trained in a distributed fashion and the results joined together by a common calibration set.

The RSCP method overall produced more efficient models compared to SCP 5 and SCP 10 and can be a good alternative to ACP when the theoretical validity of the models is an important consideration or when ACPs tendency to generate overconservative models is undesirable. However, the need to draw random samples of the available training data means that the opportunities for distributed learning are lost for RSCP.

**Fig. 6:**
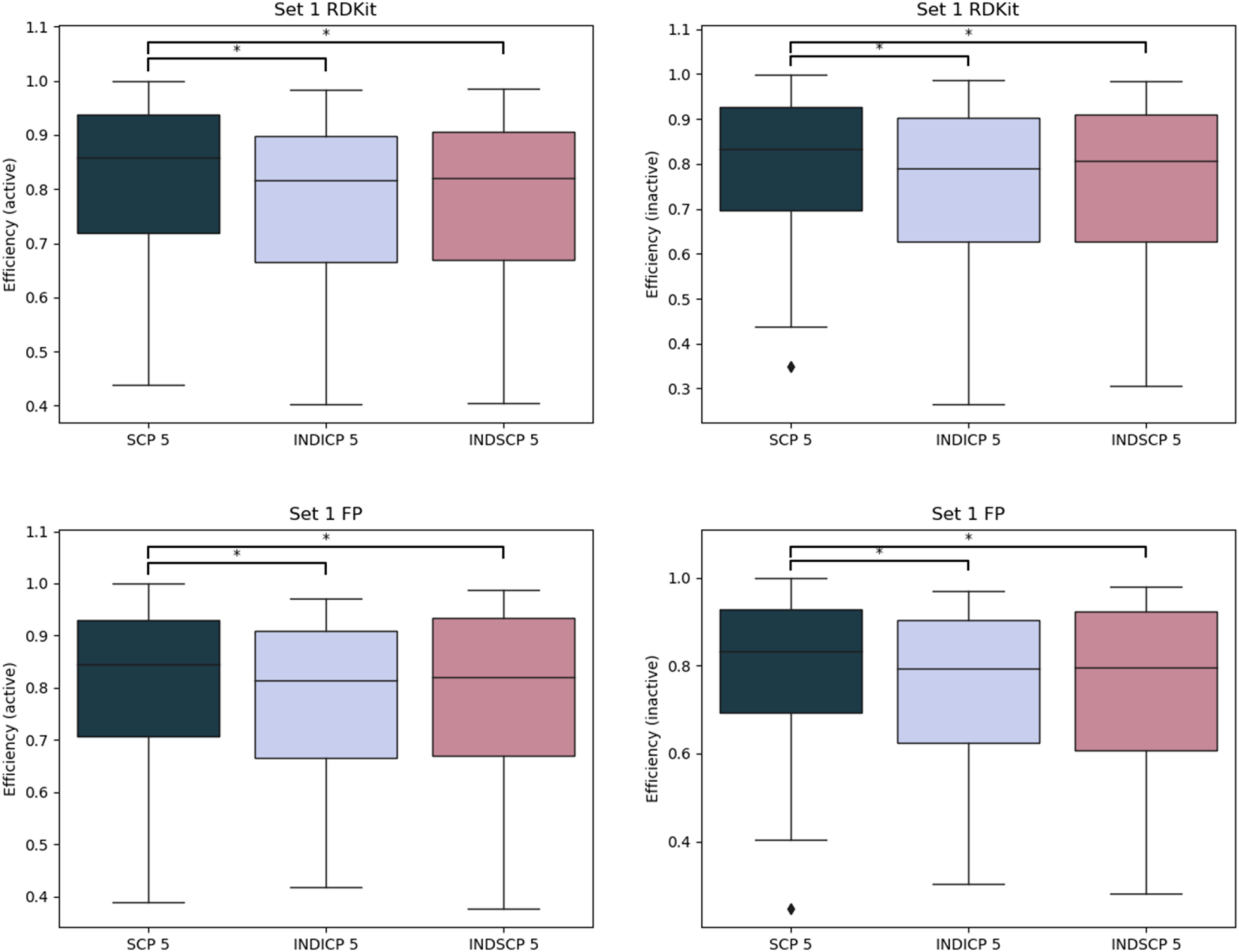
Distribution of efficiency for the individual models compared to the federated for Set 1. RDKit descriptors on top row and FP bottom, active left and inactive right. Statistically significant differences are indicated (Wilcoxon signed-rank test with Bonferroni correction for multiple testing, 0.05 significance level)

**Fig. 7:**
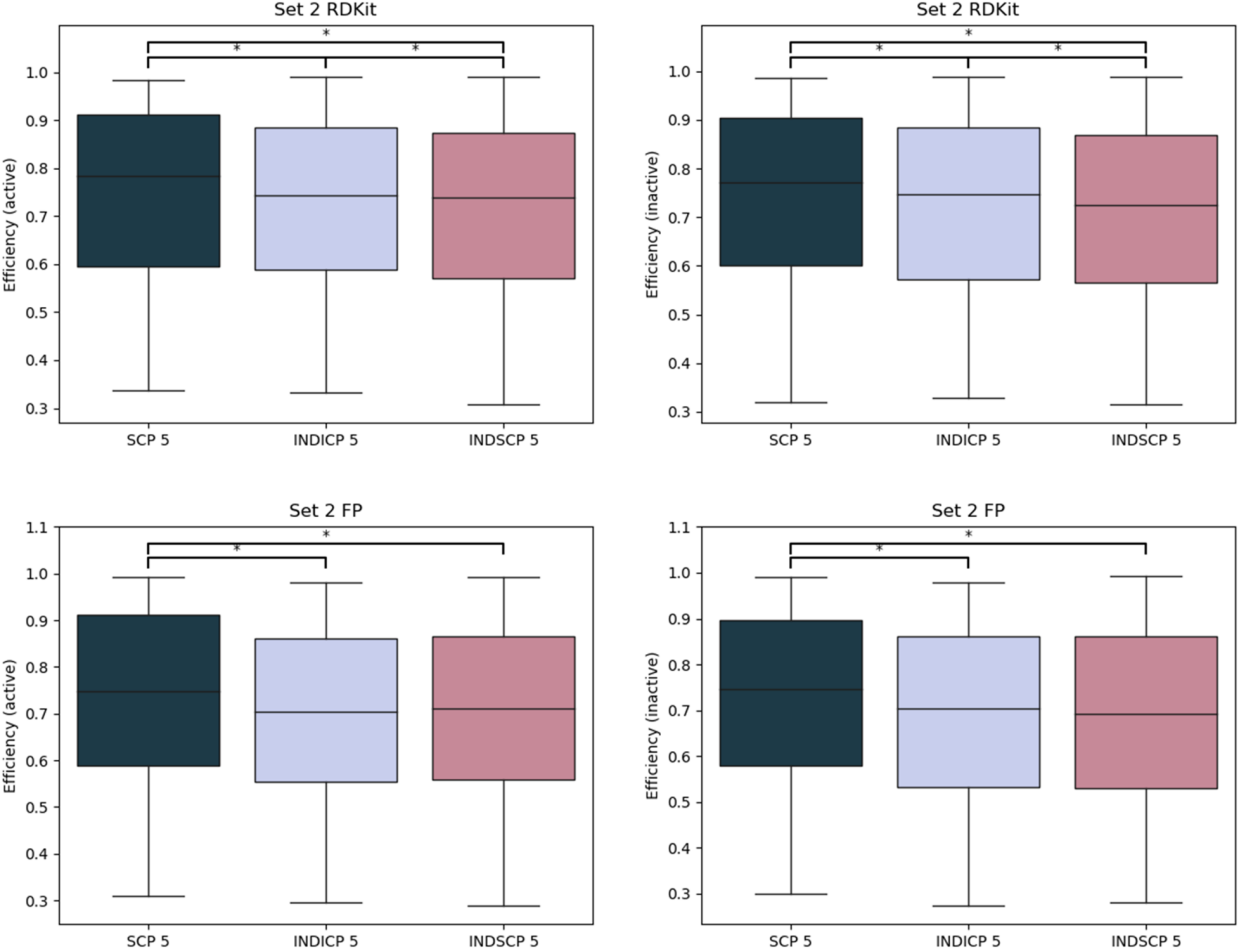
Distribution of efficiency for the individual models compared to the federated for Set 2. RDKit descriptors on top row and FP bottom, active left and inactive right. Statistically significant differences are indicated (Wilcoxon signed-rank test with Bonferroni correction for multiple testing, 0.05 significance level)

To investigate the potential utility of SCP for federated or distributed learning, we compared the results from modelling the individual parts of the training sets and using the average prediction (INDICP 5 and INDSCP 5) to the aggregated results for SCP 5. We elected to use the SCP 5 models as these had consistently better performance compared to SCP10. This reflects a scenario where data cannot be pooled to train one model and without federation the models would only have access to parts of the data, one fifth in this case. The average performance of the individual models compared to the federated model is shown in Figs. 6 and 7. Clearly, having access to more data in total improves the federated model compared to the individual models trained on only parts of the data. These results show promise for SCP for applications in federated learning. However, additional studies are required to benchmark SCP against other approaches in federated learning.

Overall, our study supports the previously published results on SCP and expand these to bioactivity prediction [15, 16]. In this study we employed Random Forest as the underlying model coupled with either molecular descriptors from RDKit or Morgan fingerprints. However, due to the flexible framework of conformal prediction any underlying method and descriptor can be used, allowing for easy conversion of already validated prediction setups. This is especially useful for federated learning since each participant can use their preferred model and descriptor type independently of what the other participants use.

## Conclusions

We have demonstrated that synergy conformal predictors can achieve predictive performance on par with ICP and ACP methods. The same type of benefit that has been observed for other Mondrian conformal predictors for heavily imbalanced data is also true for SCP and the minority class is well predicted.

Since disjoint training sets can be joined with a shared calibration set, SCP has the potential to unlock conformal prediction, and thus predictions with a defined error rate, in situations where data is difficult to aggregate for one model and for applications in federation learning. Our results indicate that good performance can be obtained from such models.

In summary, SCP is a useful addition to the conformal prediction toolbox and can complement other methods in situations where a theoretical validity is paramount or where distributed training is desired.

## Methods

### Datasets

Two different sets of data, both originating from PubChem [23], were used in this analysis and previously employed and reported on in references [21] (Set 1) and [22] (Set 2). The AID and number of compounds for each dataset is shown in Table 1. The compiled datasets both include data from AID 2314. However, differences in how these datasets were curated means that the number of compounds included is different.

**Table 1:** Datasets used in this study. Note that some of the assays deploy complex readouts that might not uniquely query the assigned target, see the full PubChem descriptions for details

AID	PubChem assay description	Inactive	Active
Set 1			
411	qHTS Assay for Inhibitors of Firefly Luciferase	68,948	1555
868	Screen for Chemicals that Inhibit the RAM Network	190,834	3545
1030	qHTS Assay for Inhibitors of Aldehyde Dehydrogenase 1 (ALDH1A1)	145,732	15,914
1460	qHTS for Inhibitors of Tau Fibril Formation, Thioflavin T Binding	45,834	1189
1721	qHTS Assay for Inhibitors of Leishmania Mexicana Pyruvate Kinase (LmPK)	289,529	1087
2314	Cycloheximide Counterscreen for Small Molecule Inhibitors of Shiga Toxin	258,344	36,955
2326	qHTS Assay for Inhibitors of Influenza NS1 Protein Function	259,823	1067
2451	qHTS Assay for Inhibitors of Fructose-1,6-bisphosphate Aldolase from Giardia Lamblia	272,893	2016
2551	qHTS for inhibitors of ROR gamma transcriptional activity	253,192	16,632
485290	qHTS Assay for Inhibitors of Tyrosyl-DNA Phosphodiesterase (TDP1)	337,970	953
485314	qHTS Assay for Inhibitors of DNA Polymerase Beta	312,599	4491
504444	Nrf2 qHTS screen for inhibitors	283,351	7406
Set 2			
1814	MLPCN Alpha-Synuclein 5'UTR—5'-UTR binding—activators	40,780	16,112
2314	Cycloheximide Counterscreen for Small Molecule Inhibitors of Shiga Toxin	30,586	26,306
2796	Luminescence-based primary cell-based high throughput screening assay to identify activators of the Aryl Hydrocarbon Receptor (AHR)	51,322	5570
463190	uHTS identification of small molecule inhibitors of tim10-1 yeast via a luminescent assay	52,443	4449
485346	uHTS for identification of Inhibitors of Mdm2/MdmX interaction in luminescent format	51,461	5431
504652	Antagonist of Human D 1 Dopamine Receptor: qHTS	50,420	6472
588726	Fluorescence-based biochemical primary high throughput screening assay to identify inhibitors of the fructose-bisphosphate aldolase (FBA) of M. tuberculosis	51,858	5034
652054	qHTS of D3 Dopamine Receptor Antagonist: qHTS	51,857	5035
687014	Luminescence-based cell-based primary high throughput screening assay to identify agonists of the DAF-12 from the parasite H. glycines (hgDAF-12)	52,572	4320
743279	qHTS for Inhibitors of Inflammasome Signaling: IL-1-beta AlphaLISA Primary Screen	47,459	9433

The chemical structures were standardized using the IMI eTOX project standardizer [24] in order to generate consistent compound representations and then further subjected to tautomer standardization using the MolVS standardizer [25]. Activity was assigned according to the PubChem annotation, and compounds with ambiguous activity were discarded.

A set of 97 physicochemical/structural feature descriptors, previous used in studies with good results [13, 26] were calculated using RDKit version 2018.09.1.0 [27]. A second descriptor set comprised of Morgan fingerprints [28] using radius 4 and hashed onto a binary feature vector of length 1,024 were also calculated using RDKit.

The data sets were randomly divided into a training set (80%) and a test set (20%).

### Study design

Four different Mondrian conformal prediction protocols (outlined in Fig. 1) were used to derive in silico models for the data sets:

1. ICP.
2. Aggregated Conformal Prediction (ACP) using 10 randomly selected pairs of *proper* training and calibration sets, respectively. (ACP 10).
3. Synergy Conformal Prediction (SCP) using a randomly selected calibration set and a random 5- or tenfold division of the *proper* training set (mutually exclusive subsets). (SCP 5 and SCP 10).
4. Synergy Conformal Prediction using a randomly selected calibration set and 10 randomly selected subsets (70%) of the *proper* training set (RSCP 10). This selection allows duplication of instances between proper training sets.

Additionally, for comparison to federated models we also use ICP and SCP on each training set separately and merged the results from the 5 parts (INDICP 5 and INDSCP 5) into one file of predicted p-values, respectively. Since the comparison, as noted above, was made to SCP5, each training set was split in 5 parts.

All underlying models were built using the RandomForestClassifier in Scikit-learn [29] version 0.20.4 with default parameters (100 estimators), that previously has been shown to be a robust and accurate methodology for bioactivity prediction [30, 31].

### Method evaluation

As introduced above, conformal predictions are typically evaluated by calculating the validity and efficiency of the predictors. In this study we define validity as the fraction of predictions that include the correct label and efficiency as the fraction of single label predictions. Since conformal predictors should be valid, focus is generally on the efficiency as a more efficient predictor will produce more useful output. For a more in-depth explanation on conformal prediction and its validation, see Norinder et al. [7].

### Statistical test

A Wilcoxon signed-rank test (significance level 0.05) with Bonferroni correction for multiple testing was used in order to determine statistical significance between the conformal prediction methods. Methods were compared across all datasets and significance levels.

## Supplementary Information

**Additional file 1.** Plots of model efficiency.

**Additional file 2.** Tabulated model results.

## Data Availability

The datasets supporting the conclusions of this article are available in the PubChem repository, see Table 1 for identifiers. Code for the conformal predictors is available from GitHub https://github.com/FredrikSvenssonUK/SCP.
