# Supplementary material for: Synergy conformal prediction applied to large-scale bioactivity datasets and in federated learning
Source: J Cheminform. 2021 Oct 2;13:77. doi: 10.1186/s13321-021-00555-7 (PMC8487527; doi:10.1186/s13321-021-00555-7)
Supplement: Supplementary file 1 — Additional file 1. Plots of model efficiency. [file 13321_2021_555_MOESM1_ESM.docx]

**Additional File 1**

**Synergy Conformal Prediction applied to Large-Scale Bioactivity Datasets and in Federated Learning**

Ulf Norinder^ꓕ,§,‡^, Ola Spjuth^ꓕ,*^, Fredrik Svensson^†,*^

^ꓕ^ Department of Pharmaceutical Biosciences, Uppsala University, Box 591, SE-75124, Uppsala Sweden

^§^ Department of Computer and Systems Sciences, Stockholm University, Box 7003, SE-164 07 Kista, Sweden

^‡^ MTM Research Centre, School of Science and Technology, Örebro University, SE-70182 Örebro, Sweden

^†^ The Alzheimer’s Research UK University College London Drug Discovery Institute, The Cruciform Building, Gower Street, London, WC1E 6BT, UK

* Ola.Spjuth@farmbio.uu.se, f.svensson@ucl.ac.uk

**Validity of predictions**

**
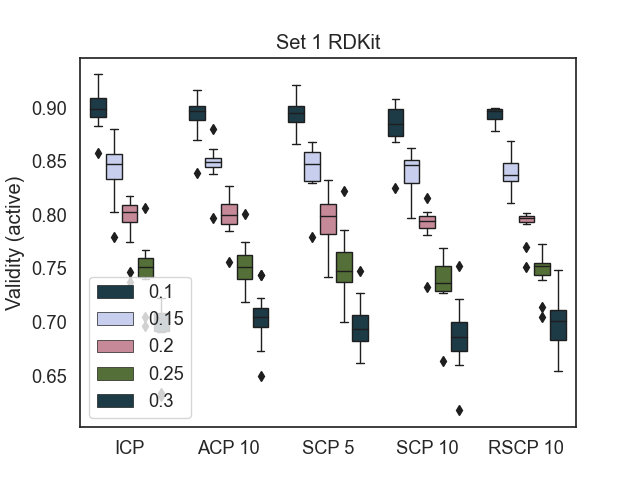

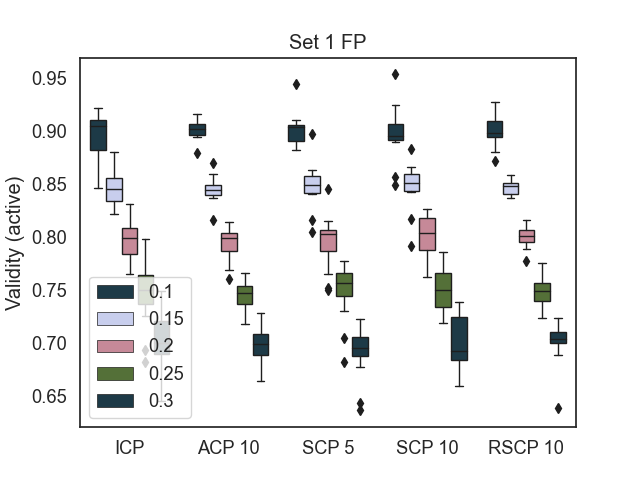
**

**Figure S1.** Validity for the active class for Set 1 using the different conformal predictors at a range of significance levels (0.1-0.3). Results for RDKit descriptors left and fingerprints right.

**
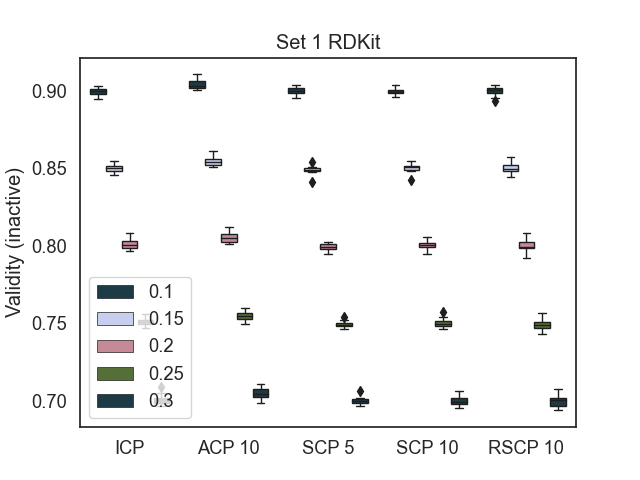

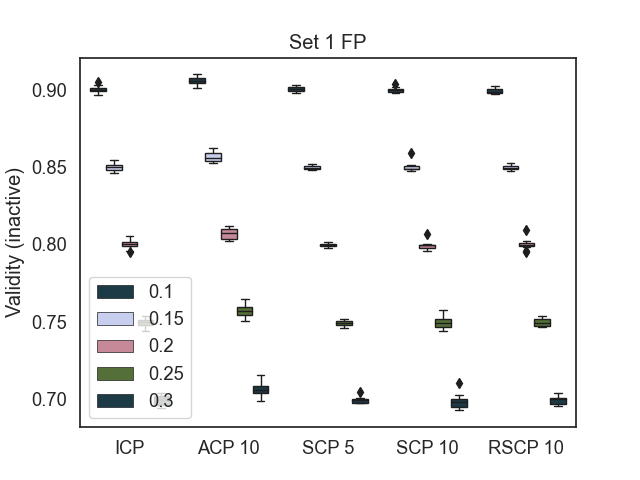
Figure S2.** Validity for the inactive class for Set 1 using the different conformal predictors at a range of significance levels (0.1-0.3). Results for RDKit descriptors left and fingerprints right.

**
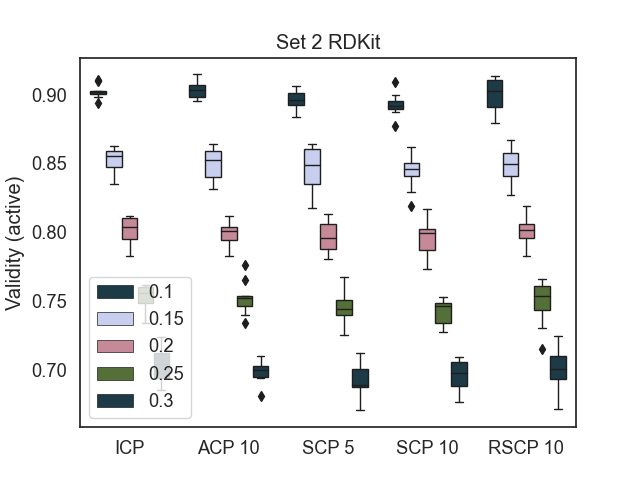

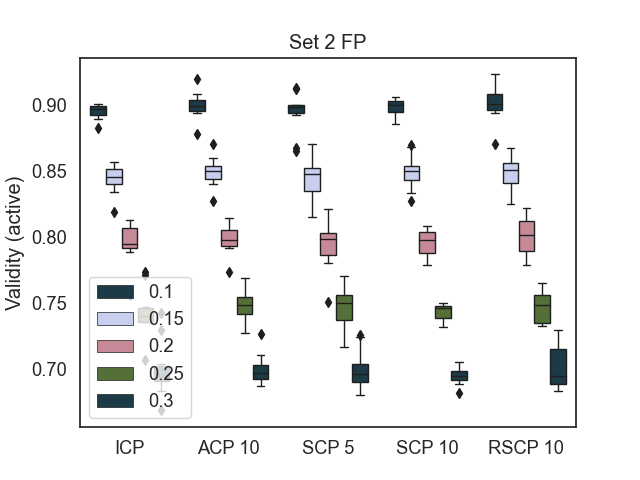
**

**Figure S3.** Validity for the active class for Set 2 using the different conformal predictors at a range of significance levels (0.1-0.3). Results for RDKit descriptors left and fingerprints right.

**
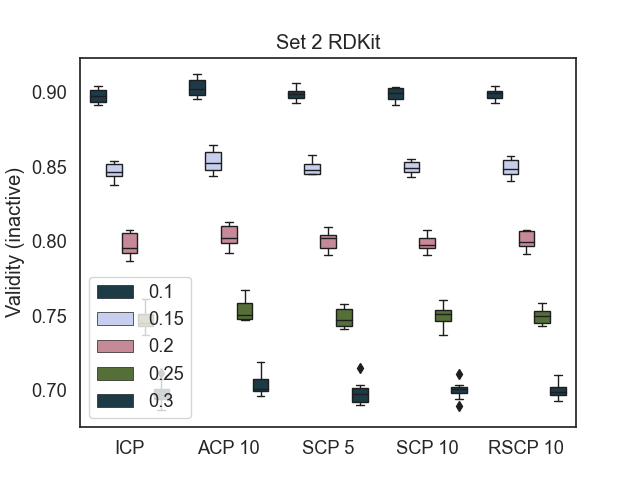

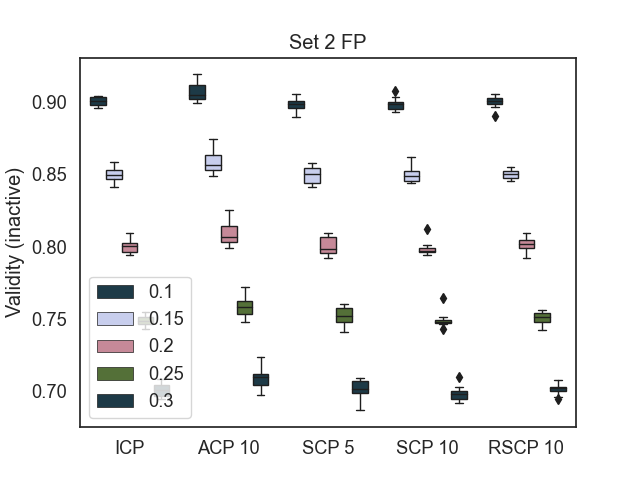
**

**Figure S4.** Validity for the inactive class for Set 2 using the different conformal predictors at a range of significance levels (0.1-0.3). Results for RDKit descriptors left and fingerprints right.
